# Jamaican Community Pharmacists-Determined Barriers to Availability of Smoking Cessation Aids

**DOI:** 10.3390/pharmacy13030081

**Published:** 2025-06-05

**Authors:** Aleena Langlay, Jeanine Abrons, Andrea Daly

**Affiliations:** 1 School of Pharmacy, College of Health Sciences, University of Technology, Jamaica, Kingston JMAAW12, Jamaica; 2 College of Pharmacy, University of Iowa, Iowa City, IA 52242, USA

**Keywords:** smoking cessation, pharmacists, barriers to sale, Jamaica, non-communicable disease, tobacco

## Abstract

Objectives: To determine the willingness of Jamaican pharmacists to stock and dispense smoking cessation aids and determine barriers to selling products. Design: A descriptive study that utilized pharmacist-completed surveys. The participants received a sectionalized survey and a structured questionnaire tool. Data collection took place over six weeks. Setting: Pharmacists practicing in pharmacies registered by the Pharmacy Council of Jamaica (PCJ) Participants: A total of fifty-seven registered community pharmacists. Results: Most pharmacies (87.7%) do not stock smoking cessation aids. The most identifiable products were nicotine patches/nicotine gum. Pharmacists’ barriers to selling were cost (42%), lack of knowledge of the process of obtaining cessation aids (27.3%), and low demand from patients/clients (22.7%). Most pharmacists (86%) were willing to stock cessation aids. Of the total product requests, 61.2% were lodged by persons 26–50 years old. The stocking of products was not independent of location (*p* < 0.005). Conclusion: The barriers to the availability of smoking cessation aids, once adequately addressed, could positively enhance the achievement of smoking cessation practices.

## 1. Introduction

It is widely documented that smoking to any extent is detrimental to individuals’ health [1]. Individuals who report lifetime smoking rates of less than one or between one and ten cigarettes per day have a greater mortality risk than individuals who have never smoked. Similar studies support that smoking one to four cigarettes per day has been linked to a more significant risk of death in both sexes and being at significantly higher risk of dying from ischemic heart disease and contributes to higher mortality from lung cancer in women [2]. Light or occasional smokers also have an increased risk of adverse health effects [3,4].

The World Health Organization (WHO) estimates that tobacco use is responsible for the death of more than 8 million people annually; approximately 22% (1.3 million) of this figure being non-smokers exposed to second-hand smoke [5]. Cigarettes contain carcinogenic compounds, increasing the risk of mutations, cell cycle dysfunction, and immune and endocrine system disruption [6]. The 34th Surgeon General’s Report found that smoking cessation is beneficial for people of all ages and can significantly reduce the risk of premature death and adverse health effects [7].

In the Caribbean region, four major non-communicable diseases (NCDs)—cardiovascular disease, diabetes, cancer, and chronic respiratory disease—have an estimated contribution to 57% all deaths in Haiti, 80% of all deaths in Jamaica, and a high of 83% in Barbados, as reported in 2018 [8]. The 2019 World Bank Development Indicators pointed to NCDs as being the leading cause of death in the Latin America and the Caribbean region, with an increase from 67% in 2000 to 76% in 2019 [9]. The Caribbean’s growing NCD burden, along with the possibility of future increases in the absence of effective prevention or control, poses a grave danger to both labor productivity and healthcare expenditures [10]. The identified risk factors of tobacco and alcohol use, physical inactivity, and unhealthy diets serve to increase the risk of dying from an NCD [11].

In 2006, PAHO supported a Caribbean-based strategy to combat NCDs, with tobacco control as a key pillar. In 2007, Caribbean Community (CARICOM) leaders took decisive action by adopting the Port-of-Spain Declaration [12], committing to the WHO Framework Convention on Tobacco Control (WHO FCTC) to implement measures reducing tobacco dependence [13]. Tobacco use is singled out as a major risk factor for non-communicable diseases (NCDs) in Jamaica. According to the Ministry of Health and Wellness (MOHW), tobacco use is responsible for 11% of all NCD deaths and 3% of communicable deaths in Jamaica [14]. The MOHW reported that 65.7% of all-cause mortality in persons five years and older was attributable to cardiovascular disease, cancer, and diabetes [15]. Of note, the prevalence of tobacco smoking in Jamaica is 17.8% among persons aged thirteen to eighteen and 14.5% in adults. This is higher than the global average of 15 percent. The MOHW has indicated that tobacco use is responsible for 6% of deaths from ischemic heart disease; 71% of cancer of the trachea, bronchus, and the lung; 10% of all lower respiratory infection deaths; and 8% of all tuberculosis deaths in persons greater than thirty years of age.

Globally, tobacco use incurs an estimated global economic cost of purchasing power parity (PPP) JMD 1852 billion (USD 1436 billion), representing 1.8% of world GDP with low and middle-income countries bearing a disproportionate burden, accounting for nearly 40% of this total figure [16]. Tobacco use has a significant economic impact on Jamaica, with an estimated 40% of a smoker’s annual income being spent on tobacco-related products [17]. The prevalence of smoking is highest among the poor or persons from lower socioeconomic backgrounds. This is because smoking is often used as a coping mechanism for stress and poverty [18], with a disproportionate prevalence observed in poor males and females in lower to middle-income countries [19]. Several interventions can be implemented to reduce tobacco use and the burden of tobacco-related disease and death in Jamaica [20]. These approaches include increasing taxes on tobacco products, banning tobacco advertising and promotion, providing smoking cessation services, and educating the public about the dangers of tobacco use.

The economic impact of treating and managing the consequences of persons who smoke is an increasing cost and drain to the budget of a country faced with relatively low income, high debt, and significant poverty rates [21]. The ability of the Caribbean region, specifically Jamaica, to secure needed advances in the overall health status of its citizens requires the focused ability to reduce NCDs and the factors contributing to them. Consequently, local healthcare team members, specifically Pharmacists, should be acutely aware of the availability of tools which can positively aid the achievement of the tobacco use reduction goals set out by CARICOM and the MOHW. This awareness can serve to aid actions to ameliorate findings indicating that, between 2010 and 2050, the mortality risks from tobacco use in many LMICs may be higher than those experienced in the developed countries of the USA or the UK between 1940 and 1980 [22].

Stopping smoking by the age of 40 reduces the extra risk of mortality by 90% for smokers; for participants who quit by the age of 30, the benefit is comparable to never having smoked initially [23]. Tobacco cessation is an essential investment in public health. In the end, it saves money while also saving lives and preserving health. The WHO FCTC came into effect in 2005 and hastened the global effort to reduce tobacco consumption. Given the potential impact that tobacco use can have on the economy of a country, the reduction of the harm on countries in the Caribbean region can have a potentially positive outcome for donor countries and those which are the recipients of large numbers of economic migrants [24].

This study examines barriers preventing pharmacists in Kingston and Saint Andrew from stocking and dispensing smoking cessation aids, despite their critical role in reducing tobacco-related morbidity and mortality. Given pharmacists’ accessibility, their involvement in smoking cessation efforts through guidance, support, and pharmacological aids is essential. However, the availability, accessibility, and willingness to provide such aids remain underexplored in Jamaica.

This research focuses on pharmacists’ perspectives regarding Nicotine Replacement Therapy (NRT) and non-NRT options, as well as the demographics of patients seeking NRT and varenicline. In engaging pharmacies in high-density areas, this study aims to provide insights into the current landscape of smoking cessation support. The findings may inform policy decisions, enhance public health initiatives, and strengthen pharmacists’ role in tobacco control efforts, ultimately reducing tobacco use in Jamaica.

### Objective of This Study

The outlined research objectives were as follows:

- To determine if Jamaican pharmacists are willing to stock and dispense smoking cessation aids.
- To determine the number of pharmacies in the parishes of Kingston and Saint Andrew that stock cessation aids and the most prevalent option provided.
- To assess the knowledge of Jamaican pharmacists on the range of smoking cessation aids available to support the process.
- To identify the pharmacist-determined barriers to the offering for sale of smoking cessation aids.
- To identify the demographics of patients requesting or using smoking cessation aids.
- To obtain the perceptions of smoking cessation advocates and regulatory authorities on the availability of smoking cessation aids and the involvement of pharmacists in the quitting process.

## 2. Materials and Methods

### 2.1. Research Design

This study utilized a descriptive, mixed method study design employing self-completed surveys and structured interviews.

### 2.2. Population and Sampling

The authorized list obtained from the Pharmacy Council (PCJ) for 2020–2021 outlined approximately 508 pharmacies registered in Jamaica. The majority of these pharmacies were community pharmacies. The sample frame consisted of a list of the registered community pharmacies in the country’s most populous area, comprising two parishes, Kingston (metropolitan) and Saint Andrew (suburban). The country’s capital, Kingston, according to The Statistical Institute of Jamaica (STATIN), houses upwards of 669,773 Jamaicans, who reside, work, and critically engage pharmaceutical services in the environs [25].

There were 130 eligible registered community pharmacies in the parishes, each with one registering pharmacist; therefore, a sample size of 98 pharmacies was initially derived using the Raosoft^®^ Sample Size Calculator (Raosoft, Inc., Washington, DC, USA), calibrated to provide a representative sample size with a five percent margin of error, 95% confidence level, and a 50% response distribution to minimize the impact of non-responses and selection bias and to ensure statistical soundness while decreasing the possible impact of biases. The sample size was increased to include all pharmacies within the geographic area, with a total of 130 pharmacists targeted [26].

This study employed purposive sampling to select advocate and regulatory entities based on their mandates in policy-making, smoking cessation, pharmaceuticals, and medical care. Representatives from four institutions were contacted via telephone and email to participate in structured interviews. Responses were sought from the key entities:

(i) The Ministry of Health and Wellness: Non-Communicable Diseases and Injuries Prevention Unit, responsible for NCD risk reduction, health promotion, disease management, surveillance, policy advocacy, and capacity building.
(ii) The National Council on Drug Abuse, which provides information on substance use and promotes treatment and prevention programs.
(iii) The Pharmaceutical Society of Jamaica, advocating for high professional standards, safe medication use, and healthcare cooperation.
(iv) The Medical Association of Jamaica, an advisory body for medical professionals on health sector reform.

Each entity was selected based on its specialized knowledge, role, and function in preventative healthcare, public health, and the delivery of healthcare to the populace. The interviews were conducted virtually, in observance of COVID-19 restrictions, with structured questions designed for completion within 20 min. The participants received the questions in advance to facilitate comprehensive responses.

The survey and interview design, time to complete same, and utilization of technology to pre-notify, deliver, provide ample response opportunities, and monitor responses were carefully managed and coordinated to obtain both qualitative and quantitative data in respect of this study. These steps were used to improve the process’s efficiency and overall effectiveness while maintaining statistical fortitude [27].

### 2.3. Inclusion Criteria

The participants were limited to pharmacists employed in the two parishes and within community pharmacies. Community pharmacy owners in the two sampled parishes, who were registered pharmacists, were also included, given their involvement with inventory control decisions inherent in their role and due to their provision of patient counseling while practicing.

### 2.4. Exclusion Criteria

Pharmacists who were not currently registered with the Pharmacy Council (PCJ) or who did not have inventory management responsibilities were also excluded. Technicians, assistants were excluded owing to the current legal restrictions that allow only registered pharmacists to dispense prescription-only drugs and provide pharmacy-only medication.

### 2.5. Measurement of Variables

The survey in Supplement Materials consisted of twelve questions, which were structured to evaluate the perceived barriers, knowledge, and demographics of patients requesting cessation aids and participants in the survey, respectively. Barriers to the offer for sale of smoking cessation aids were examined in an open-ended format to capture organic responses.

Willingness to stock smoking cessation aids was assessed at the ordinal level using several questions from the survey in the form of structured questioning utilizing Likert scales. Responses were sought in an attempt to measure how receptive pharmacists were to inventory, capital, and commercial space being allotted to smoking cessation aids. Knowledge of smoking cessation aids was assessed at the ordinal level in an attempt to determine the working knowledge Jamaican pharmacists have on the options/formulations of smoking cessation aids available. Questions from the research tool also sought to examine the variables related to the basic demographic of the patients requesting smoking cessation aids and the involvement of medical doctors in requesting the provision of these aids to their patients.

The structured interview was aimed at evaluating common themes present within the institutions/agencies responsible for the policy governing the reduction of NCDs, the provision of treatment services and options available to patients, and their own views on the barriers which may exist.

### 2.6. Data Collection and Management

The pharmacists’ tool was a sectionalized, structured questionnaire which was distributed to all 130 eligible pharmacies. The tool incorporated dichotomous questions to ascertain stocking of cessation aids, receipt of patient/professional requests, and parish of practice. Likert scales measured willingness to stock these aids. Multiple-choice questions explored the following: (i) types of NRTs stocked; (ii) perceived barriers to availability; (iii) request timelines and frequency; (iv) patient age groups requesting aids; (v) participant practice duration; and (vi) participant age. An open-ended question solicited additional comments or concerns.

The structured interview tool utilized a mix general questions and contingency questions, divided into four sections examining (i) the organizational view on the use of cessation aids; (ii) the individual views of the representatives based on mandate to include the general view on approaching smokers for participation and the possible barriers to the availability of use of cessation aids; (iii) the perception of the pharmacists’ role in introducing use of cessation aids and enabling the quitting process; (iv) the respondents awareness of national policies regarding NCD reduction and the use of cessation aids in achieving these goals and any further views on the role of pharmacists in the quitting process. The use of open-ended questions was aimed at producing a free flowing yet guided response from each respondent. The contingency questions followed those aimed at examining perceived barriers/the role of the pharmacist and were geared towards allowing respondents to expound on the particular points made in response to previously provided affirmative or dissenting responses.

Data collection was conducted over six weeks (March–April 2021). The returned surveys and transcribed interview responses were de-identified and stored on a password-protected computer system. Access was limited to only the researcher and the statistician involved in this study.

### 2.7. Reliability and Validity

The respondents were reviewed after they completed the survey to ensure that they represented the study population of registered pharmacists in the parishes of Kingston and Saint Andrew, Jamaica, and in operational community pharmacies.

The survey was piloted to test for content validity with twelve registered pharmacists working in a community pharmacy in the adjoining parish capital (Spanish Town, St. Catherine) to ensure they would not constitute a part of the sample size for the actual study. This pilot group of twelve representative pharmacists equated to 9.2% of the population (or *n* = 130 pharmacies) to which the survey was to be distributed.

### 2.8. Data Analysis

The data from the survey were analyzed using SPSS^®^ 21.0 for Windows, while univariate analysis for selected variables was undertaken to examine the central tendencies of the variables or the mean, median, and mode of the collected data from the survey tool. The chi-square test and bivariate analysis were utilized to analyze any concurrent relation between two variables or attributes from the collected data. Aggregate data only were used.

### 2.9. Ethical Considerations

IRB approval was granted by the University of Technology, Jamaica, Research Ethics Committee. The regulatory body, the Pharmacy Council, approved the use of the registered pharmacies’ and registered pharmacists’ contact information. All the participants in the survey provided consent.

## 3. Results

### 3.1. Response Rate, Distribution, Age of Respondents, Years of Experience/Practice

Registering pharmacists provided 57 responses, producing a calculated response rate of 43.84% for the survey. The participants were relatively evenly distributed based on location of practice among the two parishes, with 49.1% from Kingston and 50.9% from the parish of Saint Andrew. The review of the age of the respondents in Table 1 shows the majority (22.8%) were in the 26–30 age group, while both the 41–45 and 61 and over age groups had the next highest percentage (15.8%), respectively.

Table 2 outlines that the largest portion of pharmacists (28.1%) indicated that they have been practicing for between one and five years; 17.5% indicated that they have been practicing for over thirty-one years.

### 3.2. Knowledge of Cessation Aids

Survey participants’ awareness of available formulations of smoking cessation aids was measured with participants asked to select as many options of which they were aware. The majority of participants were aware of the nicotine patch and nicotine gum, with 36.4% and 34.4%, respectively. Additionally, 12.6% indicated awareness of the nicotine vape pen, 10.6% were aware of varenicline, and 6% indicated awareness of the nicotine nasal spray.

### 3.3. Inventory/Stocking

The participants’ responses indicated that the majority of pharmacies (87.7%) within the two parishes do not stock smoking cessation aids, while a minority of 12.3% stated that they stocked the products.

### 3.4. Reasons for Unavailability

It was noted that, of the 87.7% who did not currently stock cessation aids in their pharmacies, the majority, 68.2%, stated the absence from the inventory was due to low demand from customers, 22.7%, lack of availability from suppliers, 4.5%, lack of knowledge about smoking aids, and 2.3%, each, stated budgetary constraints and no specific reason, respectively.

### 3.5. Willingness to Include Cessation Aids in Inventory

The responses on willingness to stock cessation aids indicated that a majority (52%) were somewhat willing to stock cessation aids, while 34% stated that they were very willing. Conversely, a minority of 8% were not very willing to stock the cessation aids, while 6% of participants indicated that they were undecided.

### 3.6. Barriers Impacting the Stocking of Smoking Cessation Aids

The participants were asked to identify the barriers they believed were impacting the stocking of smoking cessation aids; participants were able to select multiple responses to this question. The majority of respondents, 42.4% (28), cited cost as the highest prohibitive factor; 27.3% stated a lack of knowledge of the process involved in obtaining the stock, while 22.7% stated low demand as a barrier to stocking cessation aids within their respective pharmacies. Further responses enumerated space (4.6%) and too much difficulty (3%) as the determined barriers to including the items in inventory.

### 3.7. Request for Smoking Cessation Aids (Patients and Medical Doctors)

**Figure 1:**
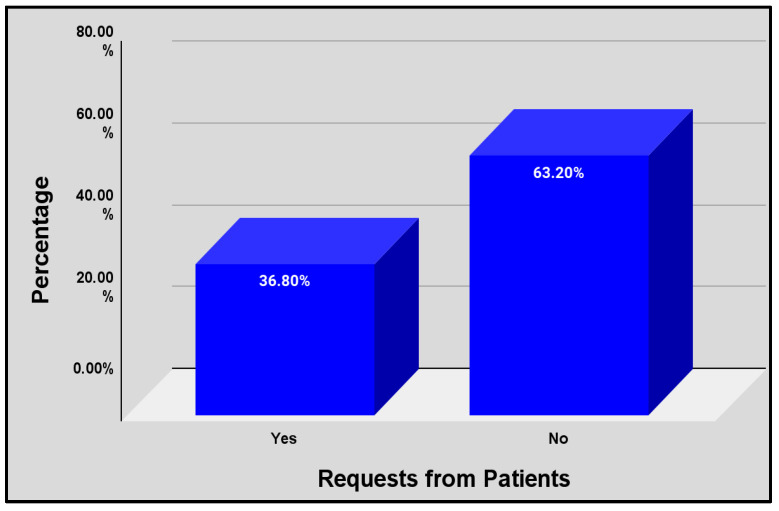
Average of requests for smoking cessation aids (patients).

The majority of pharmacists, 63.2%, stated that they did not have patients who requested cessation aids, while the minority, 36.8%, stated that they had patients requesting the aids (Figure 1).

Conversely, the minority, 19.3% of pharmacists surveyed, stated that they received inquiries/requests from medical doctors regarding cessation aids. In contrast, the majority of participants, 80.7%, stated they did not have medical doctors inquiring about cessation aids.

### 3.8. Average of Requests for Smoking Cessation Aids

The survey respondents indicated that, in the three months preceding the survey period, 54.4% had received between one to five requests for smoking cessation aids, 40.3% stated they had received no requests, and 5.3% had received between six to ten requests.

### 3.9. Gender Distribution of Patients Requesting Cessation Aids

**Figure 2:**
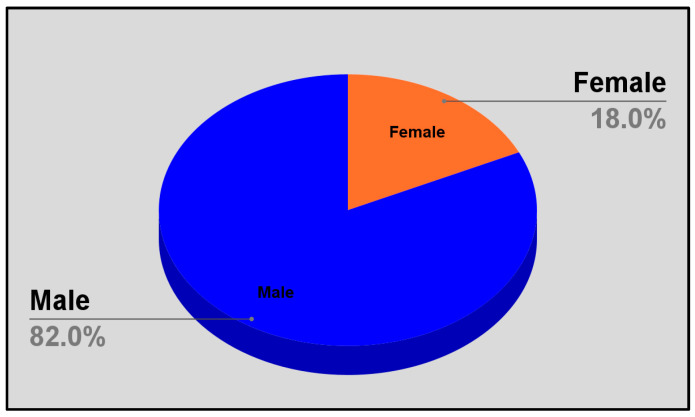
Gender distribution of patients requesting cessation aids.

The respondents indicated that the majority, 82%, of patients who requested or inquired about smoking cessation aids were men, while the minority, 18%, were women (Figure 2).

### 3.10. Age Distribution of Patients Requesting Smoking Cessation Aids

**Figure 3:**
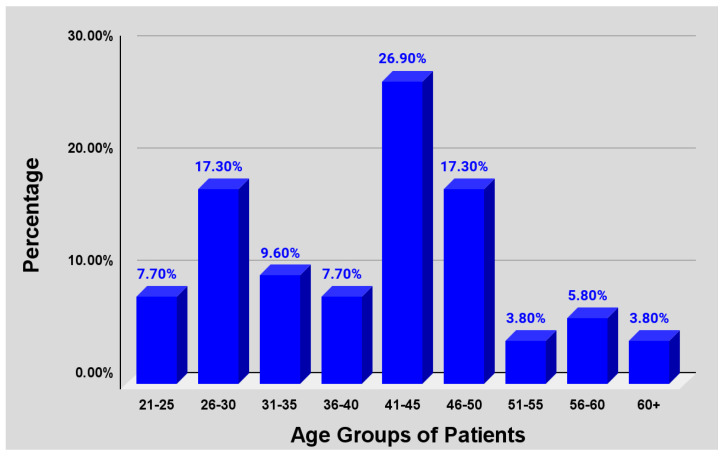
Age distribution of patients requesting smoking cessation aids.

The respondents estimated the age group distribution of persons who requested smoking cessation aids to be 26.9% in the 41–45 age group, and 17.3% from both the 46–50 age group and 26–30 (Figure 3). These groups collectively accounted for 61.2% of the age groups outlined, indicating the majority of the requests arose from patients believed to be between the ages of 26 and 50.

### 3.11. Bivariate Analysis: Location and Stocking

As outlined in Table 3, the analysis of participants stocking smoking cessation aids and the two parishes surveyed indicates that 86% of the pharmacies that stock smoking cessation aids were located in the parish of Kingston. In contrast, the remaining 14% were located in the parish of St. Andrew. From the *p*-value of 0.039, it can be deduced that there is a significant association between the location of a pharmacy and whether smoking cessation aids were stocked. The chi-square test results suggest a potential association between the variables. The Pearson chi-square (*p* = 0.039) and likelihood ratio (*p* = 0.031) show statistical significance. However, the continuity correction (*p* = 0.096) and Fisher’s Exact Test (two-sided, *p* = 0.052) indicate the result is borderline and may not be robust due to the small sample size of respondents (N = 57). The linear-by-linear association (*p* = 0.040) suggests a possible trend, but a larger sample size would be required in further research.

### 3.12. Structured Interview Response

Participation was sought from respondents (n = 4) classified as being in the advocacy and regulatory sectors, with 50% having submitted to the data collection process. The respondents were asked a series of questions pertaining to smoking cessation aids, non-communicable diseases, and the role of the pharmacist. Table 4 below depicts the major themes and sub-themes which emerged from these discussions.

### 3.13. Structured Interview Findings

The respondents all indicated that smoking was viewed as a contributory factor which has a deleterious impact on non-communicable diseases and that tobacco smoking was a significant risk factor contributing to and complicating serious health conditions regionally and locally. With this understanding and the acknowledgement that tobacco use contributes to a significant proportion of deaths worldwide, the respondents explained their rationale from the angle of health promotion and the advocacy for smoking cessation aids.

All the respondents highlighted the reduction of tobacco use among the smoking population in Jamaica as a commitment by the government with an accompanying national policy geared towards attaining this objective. All the respondents expressed that the process involved in reducing the number of tobacco smokers nationally included the implementation of several strategies to mitigate and eliminate use.

The respondents unanimously agreed that an important component tied to the success of the national tobacco cessation initiative was the availability of tobacco cessation aids and drug counseling for persons desirous of quitting. To this point, one respondent highlighted that their entity’s delivery of these measures is accessible island wide—in all fourteen parishes. There are officers assigned to address the needs in each of the island’s fourteen parishes. In tandem with strategies that provide both cessation aids and drug counseling, respondents expressed the importance of referral partnerships between members of the healthcare team (specifically medical doctors and pharmacists). The respondents cumulatively indicated that, in their individual estimation, while pharmacists are able to provide some degree of assistance—whether imparting knowledge about the availability of cessation aids, information around starting the quitting process, and the legal provision of prescription medication and pharmacy-only medication—this level of care may not be sufficient if a patient is assessed and found to be suffering from a dual diagnosis. Respondents spoke as to the inter-related nature and symbiotic relationship that often exists between a dual diagnosis and the use of tobacco in some patients. Respondents indicated that the impact of the referral process would afford a willing patient with a dual diagnosis (e.g., psychiatric illness and smoking addiction) the benefit of medical management/treatment, which would ideally incorporate smoking cessation benefits.

While all the respondents recognized the utility and importance of smoking cessation aids and advocated strongly for their use among tobacco smokers, there was some discrepancy/discord as to which type(s) of cessation aids are to be offered to these patients. Of note, one respondent emphasized an organizational preference towards the promotion and advocacy for the use of non-nicotine cessation aids such as Wellbutrin^®^ (bupropion) and Chantix^®^ (varenicline), while the other respondent expressed organizational advocacy for the use of both the nicotine replacement therapies and non-nicotine replacement therapies (via prescription). The nicotine patch and gum were identified by both respondents as the best known among patients they have interacted with over their years of practice/experience. All respondents, however, agreed that cessation aids on offer in pharmacies or via referral to a prescribing physician should be regulated cessation aids and should be offered by trained providers in a medical setting.

In response to the perceived barriers to the use of cessation aids, the most commonly identified factors stated were cost, availability, and accessibility of the non-nicotine aids through prescriptions only. This would, in their estimation, delay the person determined to abruptly quit or who would have been counseled and convinced to immediately follow through with engaging in tobacco cessation.

The respondents indicated that pharmacists’ ability based on available time or competence to screen patients adequately and efficiently could be another barrier to the provision of cessation aids and the wide-scale integration of pharmacists in the quitting process.

The need for additional training for the pharmacists was unanimously identified as necessary and important to aid in the screening of patients; training specific to tobacco cessation and integration into continuous development was identified as necessary.

All the respondents expressed that patients should be asked about smoking habits and tobacco use. It was felt that this level of screening allowed for the provision of the best possible treatment plans and the evaluation of the information to be shared with patients and the willingness of the patient to participate in the quitting process. The respondents indicated that they believe the interconnectedness of screening, patient disclosure, and pharmacist response would positively aid the health outcomes of the patients who engage the services on offer.

It was a commonly held view among the respondents that the proximity of pharmacists to patients and their relationship with the community made their role integral in promoting tobacco cessation and the provision of cessation aids. The frequency of the pharmacists’ interaction with patients, the professional being viewed as trustworthy, readily accessible due to their community location, and available to patients requiring medication and/or information, were outlined as factors that solidified their role in the quitting process. Additionally, pharmacists’ inherent knowledge of nicotine replacement/non-nicotine cessation aids by virtue of their training and practice, as well as other support that a patient may require, was seen to place them in an advantageous position to positively influence a patient’s quitting process.

The provision of pharmacist-facilitated quitting groups within the community was raised by one respondent and viewed as an opportunity where they can engender commitment of users to the quitting process, while providing an environment of support through collaboration with other stakeholders (e.g., physicians, cessation counsellors). The respondents indicated strongly that the quitting process required behavior transformation on the part of the patient and, as such, each patient’s willingness to participate in the process was of immense importance. This willingness to quit on the part of the patient could then be adequately cultivated through the provision of information and the advocacy of cessation aids on the part of the pharmacists. In the absence of this, it was thought that the pharmacists’ efforts may very well be unimpactful. In order to foster this agency among tobacco users, the respondents all expressed that there must be the provision of continuous education about the impact of tobacco use and its implication on patient’s health, especially in the presence of non-communicable diseases such as hypertension, diabetes, or cancer.

Unanimously, the respondents recommended increased availability of informational material such as pamphlets, leaflets, and in-pharmacy educational days/sessions for patients accessing pharmacy services. All the respondents highlighted the need for pharmacists to encourage the use of regulated/approved cessation aids either through provision of pharmacy-only products or in recommending and advising medical professionals on initiating or responding to referrals. The need for targeted follow-ups with patient referrals to physicians and existing smoking cessation services in cases where patients are willing to engage in the quitting process was also suggested by the respondents, and for pharmacists to actively seek collaboration with other organizations and healthcare professionals involved in the quitting process.

## 4. Discussion

Tobacco smoking is a major modifiable risk factor for non-communicable diseases (NCDs) and remains a significant public health concern in Jamaica. Unlike many other contributors to NCDs, tobacco use is identifiable and amenable to intervention. Its impact on disease burden, individual health outcomes, and national healthcare expenditure has been well-documented. In response, the WHO, PAHO, and CARICOM have led regional initiatives, while the MOHW has implemented local policies aimed at reducing tobacco use. These programs focus on prevention, cessation support, provision of cessation aids, and the training of healthcare personnel. This study explores the availability of tobacco cessation aids and the pharmacist-identified barriers.

### 4.1. Response Rate and Impact of COVID-19

The response rate was found to be in line with the accepted norm; response rates for surveys have been seen to vary between a low of 30% and a high of 61.5% [28]. Low response rates can increase the risk of response bias or non-response error being introduced. However, this can be mitigated by using survey methods to optimize response rates [29]; hence, the actions undertaken in the data collection aspect by the researchers. The low response rate prompted the researchers to employ an array of mitigating measures to increase the submission of responses, wherein the tool constructed from defined research questions was piloted and the sample size calculator along with the eligibility criteria were also accurately applied. Data collection using the structured interview tool was hindered by challenges in scheduling and obtaining consent from key stakeholders. Despite the non-response from targeted respondents, valuable insights were gained from The National Council on Drug Abuse (NCDA) and The Pharmaceutical Society of Jamaica (PSJ), which provided critical feedback on the availability of smoking cessation aids and the perceived role of pharmacists in the quitting process. This transparent representation of the findings allows for future examination of the subject matter without the impact of an ongoing pandemic and the consequent governmental restrictions on non-essential human contact.

### 4.2. Willingness to Stock and Dispense Smoking Cessation Aids

It was found that the majority of registered pharmacists in community pharmacies across Kingston and Saint Andrew were, to varying degrees, willing to stock and dispense smoking cessation aids, with only a minority expressing unwillingness or indecision. This finding suggests a favorable foundation for expanding access to cessation aids through community pharmacies, supported by the willingness of pharmacists to incorporate such products in their inventory. Previous studies have shown that physicians often fail to promote cessation interventions, even when patients could benefit, highlighting a potential gap in service delivery [30,31,32]. Pharmacist willingness was not found to be a major barrier, suggesting that pharmacists could play a valuable role in facilitating access to cessation aids and supporting broader quitting initiatives. This aligns with prior studies, where pharmacist-led cessation programs proved feasible. The recommendations from these studies suggest the viable utility of enhanced pharmacist training, private consultation spaces, and interdisciplinary collaboration to improve program effectiveness, strategies that may also be applicable in the Jamaican practice environment [33,34]. The findings of this study are also in line with the previous literature, which has demonstrated the readiness and willingness of pharmacists to provide smoking cessation services [35]. This expressed willingness starkly contrasted with data indicating that the majority of pharmacies within the two surveyed parishes do not stock smoking cessation aids. Despite the willingness of the respondents, the barriers identified have clearly impacted the ability to execute in this area. Further, the analysis of a relationship between the pharmacy’s location and the presence of the cessation aids in the inventory indicated that a significant majority of the locations that stocked smoking cessation aids were located in the metropolitan parish of Kingston.

### 4.3. Determination of Stocking Pharmacies and Prevalent Options

The majority of pharmacies in the metropolitan parish of Kingston were willing to stock cessation aids, and this finding was supported by the data presented in Table 3, showing a *p*-value of 0.039, indicating a significant statistical association between the location of a pharmacy and the availability of cessation aids in the inventory. It provides an admittedly skewed availability which favors persons residing in the parish of Kingston. Statistically, these residents would have a greater chance of obtaining cessation aids in their geographic locale. This relationship, conversely, places persons residing in the parish of St. Andrew at a statistical disadvantage, with none of the pharmacies in that parish offering these cessation aids in their inventory [35]. It should be noted, as supported by STATIN data, however, that a significant portion of the country’s population resides in the geographic area utilized for this study.

### 4.4. Relative Knowledge and Range of Cessation Aids Available

The survey respondents were most familiar with both the nicotine patch and gum formulations; this is significant, given that current legislation permits only the gum to be sold as a pharmacy-only medication, while the patch requires a prescription. The enumeration of unregulated nicotine vape pens (e-cigarettes) as being sold in non-pharmacy retail outlets was concerning. The growing use of e-cigarettes, particularly among the young, has been linked to increased risks of pulmonary disease and potential cannabis dependence, highlighting the need for stricter regulatory oversight and targeted public health responses. Despite limited research, e-cigarettes are often perceived as a safer alternative to smoking, with concerns about renormalizing smoking behaviors and increasing drug consumption [36]. A minority of respondents identified the non-nicotine replacement product varenicline as an option. The presence of this option provides an alternative tool, as the literature outlines that, regardless of the type of NRT utilized, the impact on the quitting process is a net increase in the chances of successful smoking cessation [37]. When utilized with behavioral therapy or support nicotine replacement, e-cigarettes were also seen to be more effective for smoking cessation than conventional nicotine-replacement therapies utilized in the same manner [38]. It can be argued that there is a place in therapy for the practical use of these nicotine-replacement devices or non-nicotine products, where patients have an expressed preference.

### 4.5. Pharmacist-Determined Barriers

Among non-stocking survey respondents, the majority cited low customer demand as the primary reason, suggesting a possible gap in patient awareness or professional promotion, particularly by pharmacists. Some respondents also attributed non-stocking to limited supplier availability, a notable issue given Jamaica’s reliance on imported cessation products and lack of local manufacturing. The interview respondents emphasized the need for enhanced public education utilizing materials such as pamphlets, in-pharmacy sessions, and professional engagement. The findings point to a complex interplay between low demand and limited supply, with both potentially reinforcing each other. The role of the pharmacist as the authority on medications and as an educator was put squarely at the forefront of providing the general public with information on the availability (and use) of these products. Pharmacists highlighted their role in informing patients on the use of over-the-counter nicotine replacement therapies and in collaborating with physicians on prescription-based interventions. Despite many pharmacists reporting no recent patient or physician inquiries, most had received at least some requests within the previous three months, indicating an unmet need within the surveyed population. Only a small number of participants cited lack of knowledge or budgetary constraints as reasons for not stocking cessation aids, suggesting that cost may be more a perceived than an actual barrier. Cost was reported as a limitation to providing these aids, followed by uncertainty about the process and continued low demand. These findings suggest that increasing supply, potentially lowering prices, and investing in demand-generating strategies such as education and promotion could help bridge current gaps in access and use. This dynamic could also beneficially increase the demand by persons informed/educated by pharmacists, which would remove a significant barrier [39,40]. The survey findings align with interview responses in respect of cost as a major concern and underscored the need for improved competency among healthcare practitioners, including pharmacists, in identifying patients who could benefit from cessation aids.

### 4.6. Reported Demographics of Patients Requesting/Using Smoking Cessation Aids

Consistent with the literature, the survey respondents reported that the majority of patients inquiring about smoking cessation aids were men, with women comprising a smaller proportion. This aligns with national data indicating a higher smoking prevalence among men compared to women. Given that a significant portion of the Jamaican population remains unaware of underlying conditions such as hypertension and diabetes, tobacco use in this context likely exacerbates disease progression and negatively impacts health outcomes. Regional mortality data further reinforce this concern, which is underscored by studies pointing to males being at greater risk of developing NCDs and, alarmingly, being unaware of their health status [41]. The predominance of male smokers seeking cessation support suggests that targeted interventions within this demographic could yield substantial public health benefits. Survey respondents reported that the majority of individuals seeking smoking cessation aids were estimated to be between 26 and 50 years of age, highlighting another key demographic for targeted intervention efforts. This is of concern based on the data published by STATIN outlining the estimated total labor force by age group cohort; the total labor force was estimated to comprise a majority of men between the cumulative age group of 25–54. Of this figure, more than two-thirds were being engaged productively in the employment sector [42]. This highlights the potential economic impact of smoking and, conversely, smoking cessation on Jamaica’s working-age population. Enhancing the availability of cessation aids in pharmacies could therefore have meaningful implications for workforce productivity and the broader economy. This is particularly relevant given research showing that smoking adversely affects both public health and economic stability [43].

### 4.7. Perceptions of Smoking Cessation Advocates and Regulatory Authorities

The interview respondents recognized smoking as a major contributor to the prevalence and impact of non-communicable diseases locally. Regulatory and advocacy participants emphasized the importance of smoking cessation aids and strongly supported their use among tobacco users. This aligns with national policy objectives and reflects governmental efforts to reduce tobacco use, supported by regional commitments from PAHO and CARICOM. The role of drug counseling also emerged as a theme, consistent with local pharmacy practices, which require pharmacists to counsel patients on all medication use. Previous research suggests that cessation aids, when accompanied by counseling, are more effective in achieving smoking cessation outcomes [44]. Studies have also pointed to the effectiveness of these healthcare workers in delivering smoking cessation programs and interventions [45]. Pharmacists were found to produce quantifiable results in promoting and achieving quitting success [46]. A majority of registered pharmacists in community pharmacies willing to provide cessation aids, coupled with counseling, suggests a significant potential impact on patients’ quitting success. This approach, supported by targeted training as advocated by respondents, represents a logical progression in enhancing the quitting process. Respondents recognized the island-wide presence of pharmacists and pharmacy services as a key asset to both the quitting process and implementing national policy. This accessibility was seen as enhancing the role of pharmacists in providing cessation aids and screening patients who may require them. A central theme was the importance of referral partnerships, particularly for patients with dual diagnoses, which regulatory and advocacy respondents viewed as common in practice. Implementing a structured referral process was seen as essential for ensuring patients received appropriate medical management and targeted treatment. This interconnected approach supports the expanded role of pharmacists in smoking cessation, though within defined boundaries, as determined through ongoing dialogue between pharmacy professionals, regulatory bodies, and the broader healthcare team.

The barriers identified by respondents included the cost and availability of cessation products, legal restrictions on non-nicotine aids requiring prescriptions, and the need for enhanced pharmacist training to effectively screen patients. Time constraints were noted as a challenge to implementing these interventions effectively. These barriers, along with survey findings, suggest potential obstacles to the successful integration of smoking cessation aids into healthcare practices, threatening both local and regional efforts to reduce tobacco use and mitigate associated NCD complications. It should be noted, however, that these challenges could be mitigated by the identified advantages of pharmacist involvement, particularly within community settings. Pharmacists can improve patient screening, facilitate referrals to healthcare professionals and cessation services, and capitalize on their trusted community roles. Respondents emphasized that intervention success depends on patient willingness, highlighting the need for comprehensive training in assessing readiness and delivering cessation aids. Research indicates that community pharmacist-led cessation programs have yielded positive smoking cessation outcomes, even when implemented outside formal structures [47,48]. These findings are also supported by more recent studies pointing to the efficacy and proven results seen with pharmacists’ involvement in intervention programs [46,49,50]. The skill of gauging patients’ willingness will become a key factor in the recommended screening process. The provision and availability of the outlined smoking cessation aid is key to affording patients the ability to initiate and achieve a successful quitting attempt; studies indicate that healthcare professionals can contribute positively to this smoking cessation process [51]. This is further borne out by the studies conducted in the United Kingdom, where community pharmacists had been recruited to offer smoking cessation services and aids [52], and the integration of community pharmacy services into public health programs enhanced the ability to achieve the reach of these programs [53]; this correlates with findings from France that these programs produced the required outcome of tobacco use cessation [33].

In making recommendations to overcome the barriers identified, the interview respondents recommended expanding informational resources, hosting in-pharmacy educational sessions, encouraging the use of approved cessation aids, ensuring targeted follow-ups with physician referrals, and fostering collaboration with other healthcare professionals. These recommendations align with the literature, which highlights the effectiveness of integrating community pharmacy services into public health programs to enhance their reach [53]. It also further supports the ability of the pharmacist to be effective in supporting these quitting programs [46] and in filling the expanding roles of clinical practitioners [49]. Research highlights the significant impact of cessation aids, with NRTs increasing quitting rates substantially, effective across various settings and levels of support [37]. Addressing the lack of cessation aids in pharmacy inventories could strengthen regional and local policies aimed at reducing the impact of tobacco use on NCDs in productive populations.

### 4.8. Limitations

The less-than-ideal response rate was of concern for the survey tool. The steps taken to mitigate the potential impact of the COVID-19 pandemic did not achieve the average goal of between 50 and 60% percent [26]. Mitigation strategies included converting the data collection process to a digital platform, in line with government-mandated restrictions on the activities hours of business operations and gathering-limits on persons within business places [26]. The survey tool was disseminated via email and WhatsApp, with controlled telephone follow-ups to prompt timely completion, ensuring ease of access and availability for queries during the consent and survey process. Data collection through structured interviews involved initial telephone outreach, followed by email requests for participation and consent forms. Due to COVID-19 protocols, modifications included incorporating interview questions in emails, with follow-up calls and visits where feasible. Despite efforts, response rates to the data collection tool fell short of the target, limiting the generalizability of the findings. However, the results suggest pathways for further research. The lack of input from key medical and government policy/regulatory respondents may also limit understanding of potential collaborations with the pharmacy profession. The limited response regarding MOHW policies, cessation aid availability, and pharmacists’ roles further constrains the findings. A more comprehensive thematic analysis could have emerged with greater participation from the targeted groups. Future research should aim to capture a broader cohort. Despite all the eligible community pharmacies in Kingston and Saint Andrew being included, the low response rate remains a limitation. In a bid to ensure that the impact of non-responses was kept to a minimum and to ensure statistical soundness while decreasing the possible impact of biases [26], the sample size was maintained at all the eligible pharmacies and would have been statistically ideal [28]. This study does not delve into the economic considerations of stocking smoking cessation aids, which may be a critical factor in the availability and accessibility of these products. The potential financial implications for pharmacies such as costs associated with stocking nicotine replacement therapies (NRTs) and other cessation aids were not explored in detail. Given the importance of cost in influencing pharmacy inventory decisions, particularly in community pharmacies, future research should consider incorporating economic factors, such as pricing, reimbursement policies, and profitability, to better understand their potential impact on the widespread availability of cessation aids. Having regard to these limitations, the key findings do offer valuable insights and lay the groundwork for further local and regional research. Research on pharmacists and smoking cessation aids/programs is notably underexplored in the Caribbean and Jamaica. Future research could focus on examining barriers to the availability of cessation aids locally or exploring strategies to address these challenges. This is particularly relevant within the context of the need to widely promote and make cessation aids accessible, in line with WHO, PAHO, and CARICOM policies.

## 5. Conclusions

This research highlights that a majority of registered pharmacists in the parishes of Kingston and Saint Andrew, Jamaica, are willing to offer smoking cessation aids, despite identified barriers. A significant gap in the availability of cessation aids was observed, with Kingston showing a higher number of pharmacies providing these aids. The need for more affordable options was evident, particularly given the dominance of nicotine replacement therapies (NRTs), such as gum and patches. The aversion to NRTs by a key stakeholder warrants further investigation, especially within the regulatory and advocacy sectors. The demographic most impacted by smoking cessation efforts is men, particularly those aged 25–50, who make up the majority of the employed and productive population. This group stands to benefit significantly from smoking cessation, both in terms of quality of life and economic impact.

### Recommendations

The availability of various NRT formulations and non-nicotine replacements locally should be addressed with the relevant stakeholders to expand the available options. The findings underscore the potential for enhancing pharmacist services and their impact on smoking cessation efforts. Expanding pharmacists’ roles within community health initiatives, as seen in other regions, could provide a model for promoting cessation aids. Pharmacists’ service time could be effectively utilized to promote smoking cessation by providing informational materials and offering in-pharmacy educational sessions. These initiatives would further integrate smoking cessation aids into pharmacy services and expand pharmacists’ roles in local and national health initiatives. Pharmacists’ ethical and legal duty to provide evidence-based, regulated therapies reinforces the need for using approved cessation aids. Their role as patient advocates is crucial in assessing patients’ readiness to quit and ensuring appropriate follow-up through referrals to physicians or smoking cessation services, particularly for non-compliant patients or those with dual diagnoses. A pharmacist-driven cessation program, spearheaded by the PSJ, could accelerate pharmacists’ involvement in national tobacco cessation goals. By empowering pharmacists as key outreach partners for the NCDA, their trusted status could enhance outreach efforts. Implementing these recommendations could result in significant health improvements for the Jamaican public and serve as a model for other Caribbean nations addressing the challenges of tobacco use.

## Figures and Tables

**Table 1:** Age of respondents.

Age Group Distribution	Percent
26–30	22.8
31–35	12.3
36–40	7.0
41–45	15.8
46–50	10.5
51–55	12.3
56–60	3.5
61 and over	15.8

**Table 2:** Years of practice.

Years of Pharmacy Practice	Percent
1–5	28.1
6–10	8.8
11–15	10.5
16–20	14.0
21–25	12.3
26–30	8.8
31 and over	17.5

**Table 3:** Cross tabulation—cessation aids and pharmacy location.

		Parishes				Total
		Kingston		St. Andrew		
Do you stock smoking cessation aids in your pharmacy?	Yes	86%	(6)	14%	(1)	7
	No	44%	(22)	56%	(28)	50
Total		49%	(28)	51%	(29)	57

Pearson Chi-square (asymptotic, 2-sided, sig.): *p* = 0.039.

**Table 4:** Qualitative findings.

Major Themes	Sub-Themes
Smoking is a contributing factor of NCDs	Large percent of deaths worldwide as a result of NCDs
	Risk factors include tobacco use
	Smoking is a risk factor
	Noteworthy prevalence of tobacco use exists in Jamaica
Implementation of strategies to mitigate use	Availability of tobacco cessation aids
	Drug counseling
	These strategies are offered island-wide
Referral partnerships	Clients with dual diagnosis referral to physician required
Strong advocacy for the use of smoking cessation aids	Preference towards encouraging non-nicotine aids
	Use of regulated cessation aids offered by trained providers
	Support for any kind of cessation aids
	Advocacy for the use of cessation aids
	Link between encouraging the use of cessation aids and attaining healthy lifestyle
	Negative effects of smoking such as organ damage
	Advocates the offering of cessation aids
Importance of screening for smoking habits	Disclosure of smoking habits dictates treatment plan
	Disclosure facilitates best possible health outcome for patient
	Screening and disclosure of information is inter-related with care and information provided by pharmacists
Utility of cessation aids	Cessation aids are important in quitting process
	The impact of cessation aids is predicated on the patient’s willingness to change
	Change requires patient education and knowledge of the impact of smoking
Barriers	Cost
	Availability
	Accessible through prescription only
	Need for the strengthening of training for pharmacists and other healthcare professionals
	Competency of health practitioners to screen patients adequately
The role of pharmacists in the quitting process	It is important that they interact with patients on a frequent basis

## Data Availability

The datasets presented in this article are not readily available because the data are part of an ongoing study. Requests to access the datasets should be directed to aleenalanglay@gmail.com.

## Supplementary Materials

The following supporting information can be downloaded at: https://www.mdpi.com/article/10.3390/pharmacy13030081/s1, S1: Community Pharmacy Survey.
