# Supplementary material for: Jamaican Community Pharmacists-Determined Barriers to Availability of Smoking Cessation Aids
Source: Pharmacy (Basel). 2025 Jun 5;13(3):81. doi: 10.3390/pharmacy13030081 (PMC12196336; doi:10.3390/pharmacy13030081)
Supplement: Supplementary file 1 [file pharmacy-13-00081-s001.zip › pharmacy-3501264-supplementary.pdf]

Community Pharmacy Survey

As the Registered Pharmacist having consented to participate in this research by completing this survey, you are kindly asked to answer the following questions by ticking the appropriate boxes and writing in any additional information on the lines provided or in the margin of the document. If completing this survey electronically, please insert addition comments in the box provided at the end of the page.

Provision of Smoking Cessation Aids

1 Do you stock Smoking Cessation Aids in your pharmacy?
Yes ☐ No ☐

2 How willing are you to stock more Smoking Cessation Aids in your pharmacy?
Not willing ☐ Somewhat willing ☐ Very willing ☐ Undecided ☐

3 What would be your biggest barrier to stocking and dispensing Smoking Cessation Aids?
Too much hassle ☐ Do not know enough about it☐ Space ☐ Cost ☐
I do not believe they provide a benefit ☐ Other (please specify) ___________________

4 Indicate the types of Smoking Cessation Aids which are known to you from the list below:
Nicotine Patch ☐ Nicotine Nasal Spray ☐ Nicotine Gum ☐
Varenicline ☐ Nicotine Vape Pen ☐
Other (please specify) ________________________________

5 Do you have patients request smoking cessation aids?
Yes ☐ No ☐

6 Do Medical Doctors inquire about smoking cessation aids for their patients?
Yes ☐ No ☐

7 On average how many requests for smoking cessation aids do you get in three months?
1-5 ☐ 5-10 ☐ 10-15 ☐ 15-20 ☐ Greater than 20 ☐

8 Of the patients who inquire/request smoking cessation aids, are the majority:
Male ☐ Female ☐

9 What is the age of persons who request smoking cessation aids?
21- 25 years ☐ 26 - 30 years ☐ 31- 35 years ☐ 36 - 40 years ☐
37- 45 years ☐ 46 - 50 years ☐ 51 -55 years ☐ 56 - 60 years ☐
Over 60 years ☐

Demographics of Participant

10 In which parish do you practice?
Kingston ☐ Saint Andrew ☐

11 How long have you been a registered pharmacist in Jamaica?
1-5years ☐ 6-10 years ☐ 11- 15 years ☐ 16 - 20 years ☐
21– 25 years ☐ 26 – 30 years ☐ Greater than 30 years ☐

12 How old are you?
21- 25 years ☐ 26 - 30 years ☐ 31- 35 years ☐ 36 - 40 years ☐
41- 45 years ☐ 46 - 50 years ☐ 51 -55 years ☐ 56 - 60 years ☐
Over 60 years ☐

END OF SURVEY
